# MicroRNAs in Serum Exosomes as Circulating Biomarkers for Postmenopausal Osteoporosis

**DOI:** 10.3389/fendo.2022.819056

**Published:** 2022-03-10

**Authors:** Hongli Shi, Xin Jiang, Cuidi Xu, Qun Cheng

**Affiliations:** Department of Osteoporosis and Bone Disease, Huadong Hospital Affiliated to Fudan University, Research Section of Geriatric Metabolic Bone Disease, Shanghai Geriatric Institute, Shanghai, China

**Keywords:** exosome, miRNAs, postmenopausal osteoporosis, circulating biomarker, fragility fracture

## Abstract

Postmenopausal osteoporosis (PMOP) is the most common skeletal disease in postmenopausal women and has become a global public health issue. Emerging evidence demonstrated the important relationship between microRNAs and PMOP. However, miRNAs have not yet been reported in PMOP. Hence, the present study aimed to investigate the differences in miRNA expression profiles in PMOP with fragility fractures to identify the key circulating miRNAs in serum exosomes and to validate these molecules as potential biomarkers. Postmenopausal women with osteoporotic fracture and normal bone mass were enrolled. Serum exosomes were isolated by traditional differential ultracentrifugation from participants. Isolated exosomes were identified by electron microscopy, western blotting and nanoparticle-tracking analysis and then examined for exosomal small RNA sequencing. The expression of miRNAs was compared by sRNA deep sequencing and bioinformatics analysis. Three miRNAs (mir-324-3p, mir-766-3p and mir-1247-5p) were found to be associated with BMD of L1-L4, FN (femur neck) and TH (total hip), while mir-330-5p and mir-3124-5p were associated with BMD of FN and TH. Furthermore, mir-330-5p was found to promote the ALP activity of hBMSCs, while mir-3124-5p showed the opposite result. The results showed that serum exosomal miRNAs were differentially expressed in postmenopausal osteoporosis patients with fragility fractures. Our study provides the first evidence that exosomal miRNA profiling revealed aberrant circulating miRNA in postmenopausal osteoporosis. Mir-324-3p, mir-766-3p, mir-1247-5p, mir-330-5p and mir-3124-5p, which were associated with bone mineral density (BMD), may serve as candidate diagnostic biomarkers as well as potentially contribute to pathophysiology of PMOP.

## Introduction

Osteoporosis (OP) is a systemic bone disorder characterized by an imbalance between bone formation and resorption, which leads to a reduction in bone mass (1). In women, postmenopausal osteoporosis (PMOP) is characterized by low bone mass and consequent fragility fractures, which have impaired quality of life and increased mortality in the population (2, 3). Although the measurement of BMD by dual energy X-ray absorptiometry (DXA) has been regarded as the “gold standard” and current approaches for predicting fractures are largely based on the measurement of BMD, BMD is associated with only 30–50% patients with major fragility fractures (4). In addition, the change in bone mass by DXA is gradual, and a period of 1 or 2 years is usually necessary to identify significant changes, which is inadequate to monitor bone loss (5). It is urgent to find a more accurate way to diagnose OP and predict fracture risk.

In recent years, miRNAs have attracted extensive attention for their roles in many biological processes, such as cell proliferation, differentiation, apoptosis and migration (6–9). It is known that miRNAs in serum may be associated with biological processes and play an important role in the progression of diseases. Regarding bone metabolism, some researches have shown that miRNAs were associated with bone metabolic disorders (10) and several miRNAs were proved to regulate the osteogenic differentiation of BMSCs by mediating β-catenin-dependent migration (11) or contribute to the regulation of Smad5 (12) and Runx2 (13). Additionally, it was revealed that miRNAs might be biomarkers with diagnostic and prognostic potential in cancer and other diseases (14). However, the complexity and inherent heterogeneity of miRNAs in the circulation make it difficult to develop biomarkers and let alone evaluate the prognosis of diseases.

Exosomes are cell-derived spherical lipid bilayer vesicles (EVs) with a diameter around 40-160nm, widely present in various body fluids, carrying proteins, mRNAs and miRNAs that can be transferred from donor to recipient cells *via* target cell membrane fusion. After release, exosomes are taken up by neighboring or distant cells, and the miRNAs contained within modulate such processes as interfering with the microenvironment, facilitating proliferation, differentiation, senenscence and apoptosis. Exosome also regulate epigenetic processes by delivering miRNAs and regulate the biological function of recipient cells in bone regeneration. A number of researches have confirmed that bone marrow mesenchymal stem cells-derived exosomes could improve bone mass by promoting osteogenesis or inhibiting osteoclastogenesis through mir-196a (15), mir-150-3p (16), mir-181a (17), mir-218 (18), and mir-29a (19). More importantly, serum exosomal miRNAs may affect bone metabolism, and can be good biomarkers based on their stability under various storage conditions. Ruchun Dai et al. reported that, serum exosomes highly expressing mir-19b-3p improved the osteogenic differentiation ability by decreasing the expression of PTEN protein (20). MiRNAs in exosomes were stable enough under different storage conditions even at 4°C for a short time (21), suggesting that serum exosome miRNA panel could be as a noninvasive biomarker for the assessment of bone loss and the detection of fragility fractures. However, there are few studies on serum exosome miRNAs in osteoporosis.

In this study we tested the potential role of these molecules as biomarkers in diagnosis and prognosis of osteoporosis and fragility fracture. We compared the serum exosomal miRNAs between osteoporosis with fragility fractures and normal BMD without fracture in postmenopausal women, to dissect the links between serum exosomal miRNAs and severe osteoporosis. The study of miRNA signatures will provide a deeper understanding of bone turnover mechanism to further identify potential diagnostic biomarkers of fragility fracture and assess fracture risk.

## Materials and Methods

### Patient Serum Samples

A total of 577 postmenopausal women aged 65-75 years from two communities were enrolled and history of fragility fractures were collected, BMD of the lumbar, vertebra and hip were detected by DXA. Hip fractures and spine fractures were verified by review of medical records and imaging examinations. According to the National Osteoporosis Foundation, fragility fractures are fractures resulting from any fall from a standing height or less (22). All the participants were divided into two groups: subjects of control group (CON) had no fracture history with T-score of BMD of any site >-1.0, while subjects of severe osteoporosis group (SOP) suffered from fragility fractures in the vertebral spine and/or hip with BMD T-score of any site ≤-2.5. Serum levels of calcium, phosphorus, 25-hydroxyvitamin D(25(OH)D), parathyroid hormone (PTH), Procollagen 1 N-Terminal Propeptide (P1NP) and β-carboxy-terminal collagen crosslinks (β-CTX) were obtained to rule out secondary OP. Participants using insulin, sex hormones, glucocorticoids, anti-osteoporosis drugs such as bisphosphonates, estrogen and progesterone replacement, selective estrogen receptor modulators (SERMs), parathyroid gland hormones or other drugs affecting bone metabolism, or those who suffered from diabetes, severe cardiopulmonary disease, liver and kidney disease, endocrine and metabolic diseases, autoimmune diseases, malignant tumors and hyperlipemia were excluded from the study.

We implemented the following exclusion criteria: participants without consent form (n = 52), those not finishing the DXA scan (n = 37), BMD or history of fragility fracture not meeting the requirement of the study (n = 298), and those who had medical condition excluded from the study (n = 156). Finally, there were 18 participants remained in CON, and16 participants remained in SOP. This study was approved by the Medical Ethics Committee of Huadong Hospital (2019K055) and informed consent was obtained from all participants. The participants’ information was listed in **Table 1**.

**Table 1:** Characteristics of the participants in this study.

Characteristics	Control, n = 18	SOP, n = 16	*p*-value
Age (year)	67.28 ± 5.2	67.0 ± 3.2	0.86
Height (cm)	157.53 ± 5.8	153.0 ± 6.8	0.04
Weight (kg)	59.62 ± 6.7	53.51 ± 7.4	0.02
BMI (kg/m^2^)	24.02 ± 2.4	22.92 ± 3.6	0.31
Fracture of vertebra (%)	0	68.75	0.000
Fracture of hip (%)	0	43.75	0.000
Serum creatinine (umol/L)	61.8 ± 12.9	56.93 ± 8.9	0.27
AKP (U/L)	74.6 ± 17.8	80.23 ± 25.1	0.52
Serum calcium (umol/L)	2.41 ± 0.06	2.38 ± 0.11	0.53
Serum phosphorus (umol/L)	1.28 ± 0.23	1.19 ± 0.13	0.25
25(OH)D_3_ (ng/ml)	30.33 ± 15.11	28.79 ± 14.59	0.82
PTH(pg/ml)	31.8 ± 9.0	45.3 ± 20.2	0.14
β-CTX(pg/ml)	482.48 ± 209.58	476.79 ± 286.96	0.97
P1NP(ng/ml)	61.2 ± 12.4	61.1 ± 13.3	0.96
BMD of LS (g/cm^2^)	0.884 ± 0.13	0.603 ± 0.06	0.000
BMD of FN (g/cm^2^)	0.705 ± 0.11	0.511 ± 0.10	0.000
BMD of TH (g/cm^2^)	0.752 ± 0.21	0.612 ± 0.16	0.04

LS, lumbar spine; FN, femoral neck; TH, total hip.

### Serum Exosomes Isolation

For this study, blood from 34 participants were sampled on weekday mornings between June 2017 and December 2017. A 10 ml tube of whole blood was collected by the trained nurse following standard procedures using a serum separator tube (367820, BD) from each participants. Serum samples were allowed to clot for 30 minutes at room temperature, and then centrifuged at approximately 1000g for 10 minutes. 3 ml peripheral serum from each participant was collected, and exosomes were isolated from serum by traditional differential ultracentrifugation in four steps. At first, serum was diluted with sterile phosphate-buffered saline to 50ml, centrifugation at 3000×g for 30 min was performed, then supernatant was centrifuged at 12,000×g for 45min followed by ultracentrifugation for 2h at 120,000 ×g in 4°C. The exosome pellet was re-suspended in 100ul lysis buffer or sterile PBS, depending on subsequent experiments.

### Transmission Electron Microscopy (TEM)

The suspension was mixed with an equal volume of 4% paraformaldehyde, and 25ul of the solution was taken up to the loaded copper mesh, dried at room temperature for 20 minutes, and the liquid on the filter screen was blotted from one side with a filter paper, and 30ul of phosphotungstic acid solution was added, stained for 5 min at room temperature, and then was blotted with a filter paper and dried at room temperature. The exosomes were photographed under a transmission electron microscope.

### Western Blot Analysis

Exosomes were lysed in RIPA buffer with 1% phenylmethylsulfonyl fluoride (PMSF) and placed on ice for 10 minutes. Protein was quantified by using BCA protein quantitative kit (Sangon Biotech, Shanghai) according to the instruction. The concentration was adjusted by appropriate amount of radioimmunoprecipitation assay (RIPA) buffer, and sodium dodecyl sulfate (SDS) loading buffer of 1/4 volume was added. Protein samples were loaded, separated on 10% SDS-Polyacrylamide Gel Electrophoresis (SDS-PAGE), and transferred to a polyvinylidene difluoride (PVDF) membranes, followed by blocking for 1 hour in 5% non-fat skimmed milk in tris buffered saline with tween(TBST) solution. After blocking, membranes were incubated with primary antibodies against TSG101 (1:1000 dilution, ab125011, Abcam) and CD63 (1:1000 dilution, ab216130, Abcam) respectively overnight at 4°C. Membranes were then washed using TBST for three times and incubated in secondary antibody for 1hour in room temperature. At last, membranes were washed and developed by Tanon3500 gel imaging and photographing system (Tanon Science & Technology Co, Ltd.)

### Nanoparticle Tracking Analysis (NTA)

Isolated pallets were analyzed by the Nanosight NS300 System (Malvern Instruments, UK) configured with a 488 nm laser and a high sensitivity scientific CMOS camera to determine the size and quantity of particles. The exosome samples were diluted (1:300) in particle-free PBS to an acceptable concentration, according to the manufacturer recommendations. Samples were analyzed under constant flow conditions (flow rate=50) at 25°C. For bootstrapped samples, 30 s × 60 s successive videos were captured with a camera level of 16. Data were analyzed using NTA 3.1.54 software with a detection threshold of 5. For the validation cohort, 15 s × 60 s videos were captured with a camera level of 16 and a detection threshold of 10. Laser-irradiated nanoparticles are captured for 60 seconds and particle were analyzed by NTA software.

### MiRNA Library Construction and Sequencing

Serum exosomes were isolated, prepared and sent to BGI-Wuhan (Wuhan, China) for miRNA library construction and next-generation sequencing. For each sample, clean reads were obtained *via* removing the low quality readsand aligned with the human genome. Clean reads were further mapped to sRNA in the GenBank and Rfam to analyze their distribution and annotate small RNA sequences. After sequencing by an Illumina sequencer, image analysis, and base identification, the raw reads after quality control were harvested. Clean reads were aligned against known miRNA precursors and mature miRNAs in the miRBase to identify conserved miRNAs. We filtered out all the samples with library size (total uniquely mapped reads) <50,000 reads. We calculated miRNAs normalized counts by using Variance stabilization normalization (VSN). The resulting VSN counts were corrected for various cohorts along with the removal of the unwanted variances by using the R (v 3.2.2) package RUVSeq (v 1.14.0). We filtered out miRNAs that had a VSN read count less than 0.5 in the 95% of control and PMOP samples, respectively. Fold-change (FC) > 2(|log_2_FC|>1) and FDR < 0.05 were the criteria for differential expression.

### MiRNA Target Prediction and Relevant Signaling Pathway

Targets of miRNA were predicted by using Targetscan (http://www.targetscan.org) and Gene Ontology (GO) enrichment analysis of all predicted target genes was performed using DAVID online tool (https://david.ncifcrf.gov/summary.jsp). The relevant signaling pathways were analyzed using the MirPath in DIANA (http://diana.imis.athena-innovation.gr/DianaTools/index.php?r=site/page&view=software).

### Cell Cultures, Transfection and Osteogenic Differentiation

Bone Mesenchymal Stem Cells (BMSCs) were purchased from CyagenBioscience Inc and cultured in human bone marrow mesenchymal stem cell basal medium with 10% fetal bovine serum, penicillin-streptomycin and glutamine at 37°C with 5% CO_2_. Cells were transfected with 20nM microRNA mimics on day 0 and cultured in human mesenchymal stem cell osteogenic differentiation basal medium with 10% fetal bovine serum, 1%glutamine, 1%ascorbate, 0.2%β-Glycerophosphate and 0.01% dexamethasone from day1 to day7 to induce osteogenic differentiation. Mediums were changed every 2 days.

### Alkaline Phosphatase (ALP) Activity Assay

ALP activity was examined by using Alkaline Phosphatase Assay Kit (ab83369, Abcam) in bone mesenchymal stem cells on day 7 after transfecting with related miRNA mimics or vehicles. 5mg pNPP was dissolved in solution with 0.1 M glycine, pH 10.4, 1 mM MgCl_2_ and 1 mM ZnCl_2_. Cell culture medium was discarded and 100ul pNPP solution was added 15 minutes. The absorbance was examined at 405 nm.

### Statistical Analysis

Numerical data was presented as the mean ± standard deviation. Significant differences between two groups were determined by Student’s t test. Differences between multiple groups were compared using one-way analysis of variance (ANOVA) followed by Tukey’s *post hoc* test. Statistical analysis was performed using SPSS and correlations were analyzed using Spearman data. *p*<0.05 was considered statistically significant. Statistical significance is displayed as *P < 0.05, **P < 0.01, and ***P < 0.001.

## Results

### Characterization of Participants

18 participants were in control group who had a normal bone mass and16 participants were in SOP group who suffered from vertebral fracture (68.75%) and/or hip fracture (43.75%). BMI, age, biochemical markers and bone metabolism markers, 25(OH)D, PTH, and BMD between the two groups were shown in **Table 1**. No statistical differences in the age were observed between the two groups. However, the mean values of height and weight were lower in SOP than those in CON (*p*<0.05). In addition, the SOP group have significantly lower BMD at all measured sites (lumbar spine, femoral neck and total hip) compared to CON group.

### Serum Exosome Characterization

**Figure 1:**
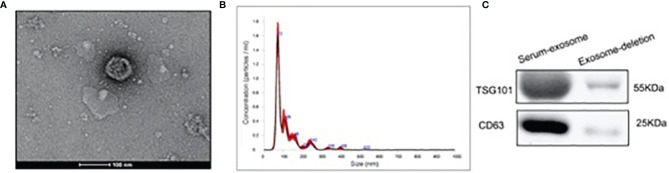
Characterization and identification of serum exosome by ultracentrifugation. **(A)** Exosomes were analyzed by electron microscopy (Scale bar=100 nm). **(B)** Size distribution of exosomes were analyzed by the Nanoparticle tracking analysis. **(C)** Western blotting was applied to detect the exosomal markers TSG101 and CD63 in serum sample after exosome isolation and exosomes isolated from serum sample.

Isolated exosomes from participants were identified by a combination of TEM, western blotting and NTA. In **Figure 1** results on exosome isolation were only shown for control group as representative for the feasibility of the method to isolate exosomes. According to TEM results, we observed that isolated particles were approximately 80 nm in diameter and appeared to be round vesicles (**Figure 1A**). To further confirm the identity of the isolated pellets as exosomes, we performed the NTA measurements and observed that the size of isolated particles was112.8 ± 2.0nm in diameter (**Figure 1B**). In detail, the size of exosome was 112.8 ± 2.0nm in CON group and 175.7 ± 5.1nm in SOP group (**Supplementary Figures 1A, B**). The concentration of exosome was 6.07e+008 ± 3.90e+007 particles/ml in CON group and 6.52e+008 ± 1.57e+007 particles/ml in SOP group (**Supplementary Figures 1C, D**). Western blotting identified increased exosome-enriched protein markers CD63 and TSG101 in isolated particle samples, compared to serum samples after exosome-isolation procedure (**Figure 1C**).

### Serum Exosome-Associated miRNAs Profile

**Figure 2:**
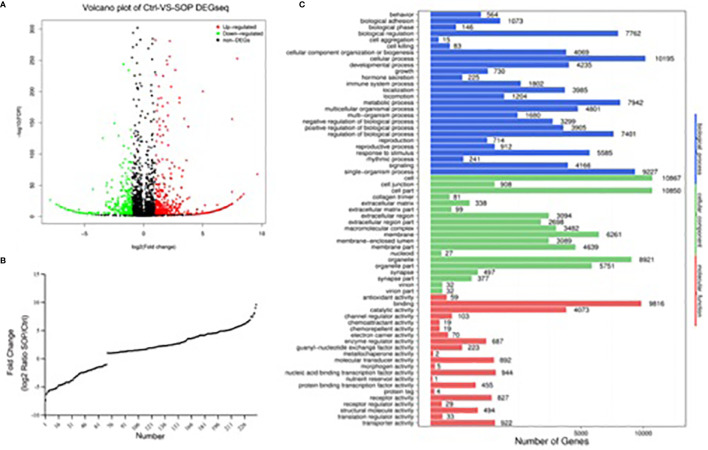
Differentially expressed miRNAs in serum exosomes between SOP and control library. **(A)** Volcano plot was applied to show differentially expressed exosomalmiRNAs in severe osteoporosis group and control group. **(B) **Deletion of unknown miRNA and diverseregulatedmiRNAs were analyzed with miRbase database. X axis shows the number of differently expressed known miRNAs, and the Y axis shows the fold change of SOP/Ctrl. **(C)** Classification of potential target genes for differently expressed known miRNAs by GO analysis. X axis shows the number of target genes, and the Y axis shows the GO terms of biological process, cellular component and molecular function.

DEGseq was used to identify differentially expressed microRNAs between CON group and SOP group by second-generation sequencing. According to Volcano Plots, there were statistically significant regulated miRNAs between CON and SOP (**Figure 2A**). We further filtered and analyzed the differentially expressed miRNAs in serum exosome with the miRBase database to obtain all known miRNA counts, and unknown miRNAs were excluded. Compared to control group, 169miRNAs were significantly upregulated (*p*-value<0.05 and log_2_FC [log_2_FC]>1) and 70miRNAs were downregulated in SOP group (**Figure 2B** and **Supplementary Table 1**).

### Gene Ontology Enrichment Analysis

To greater determine the role of differentially expressed miRNAs in pathological process of PMOP, we input predicted target genes of these known miRNAs to DAVID for GO functional analysis to understand the functional distribution characteristics. The items of biological process(BP), cellular component(CC) and molecular function(MF) terms were presented in **Figure 2C**. The top 3 significant terms from the analysis showed that in the BP category, the diverse miRNAs were involved in cellular process, single-organism process and metabolic process. For the CC category, the different miRNAs were correlated with cell, cell part and organelle. For the MF category, the diverse miRNAs were enriched in binding, catalytic activity and nucleic acid binding transcription factor activity.

### Signaling Pathway Analysis of Target Genes

**Figure 3:**
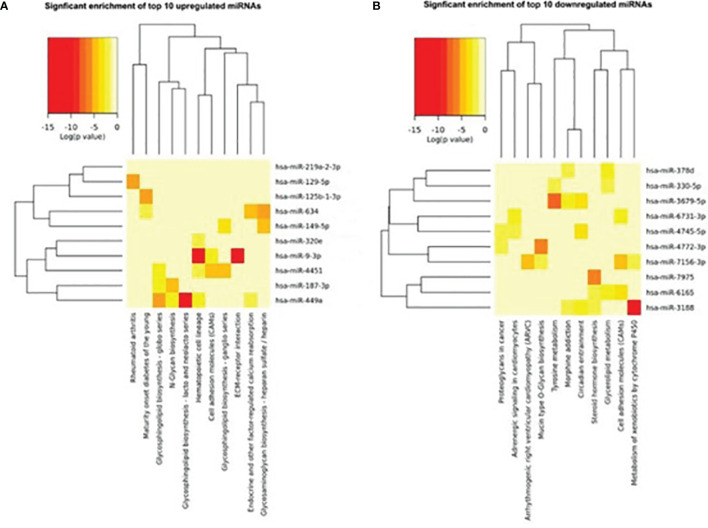
Heat map of signaling pathway enrichment for target genes of top 10 differentially expressed miRNAs. Data of top 10 upregulated **(A)** and downregulated **(B)** miRNAs were analyzed by online bioinformatics tool DIANA. Each row and column represent a miRNA and pathway respectively. The red color shades represent high relative levels and yellow shades represent lower relative levels.

We further analyzed and investigated the potential function of differently expressed top 10miRNAs with online bioinformatics data analysis tools TargetScan and DIANA. Target genes of upregulated miRNAs were mainly involved in rheumatoid arthritis, maturity onset diabetes of the young, glycosphingolipid biosynthesis-globo series, N-glycan biosynthesis, glycosphingolipid biosynthesis-lacto and neolacto series (**Figure 3A**). Target genes of downregulated top 10miRNAs were mainly involved in proteoglycans in cancer, adrenergic signaling in cardiomyocytes, arrhythmogenic right ventricular cardiomyopathy (ARVC), and mucin type O-glycan biosynthesis (**Figure 3B**).

### Correlation Analysis of miRNAs With Bone Mineral Density

**Figure 4:**
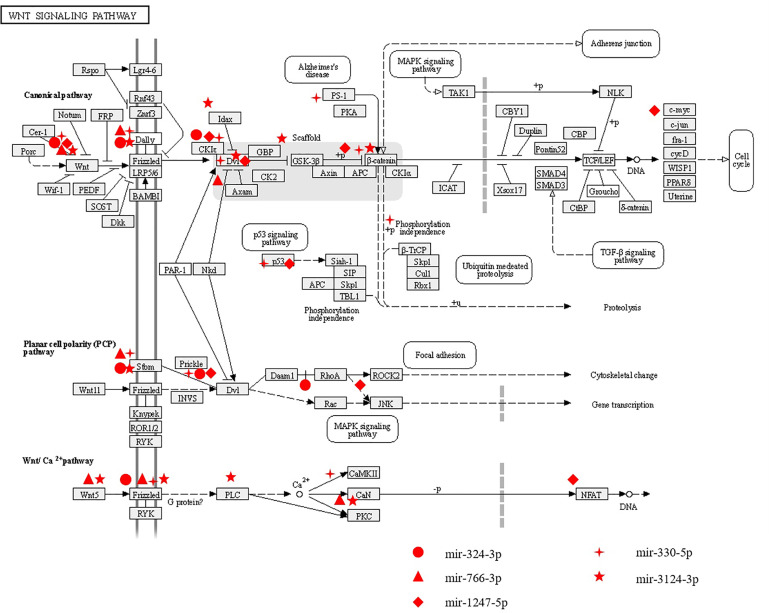
The potential roles of miRNAs related to BMD in Wnt signaling pathway. Five differently expressed miRNAs (including mir-324-3p, mir-776-3p, mir-1247-5p,mir-330-5p and mir-3124-3p) associated with BMD were predicted to play roles in Wnt signaling pathway through regulating their potential target genes.

In this study, we analyzed the correlation between miRNA profiling that was filtered with FC>2(|log2FC|>1) and p-value<0.05 and BMD so as not to miss miRNAs that may have impact on the bone metabolism.To greater confirm the core exosomal miRNAs involved in the progression of PMOP, correlations between known miRNAs and BMD of lumbar L1-L4, FN, and TH were analyzed and the results were shown in **Table 2**. Five miRNAs have been found associated with BMD of 2 or 3 areas of the bone: mir-324-3p [log_2_FC=-1.54,P<0.0001], mir-766-3p [log_2_FC=-1.3,P<0.0001], mir-1247-5p [log_2_FC=2.34,P=0.0029], mir-330-5p [log_2_FC=-5.84,P=0.002], mir-3124-3p [log_2_FC=5.72,P<0.0001].Three miRNAs were related to the BMD of L1-L4,FN and TH (mir-324-3pand mir-766-3p were positively correlated, while mir-1247-5p was negatively correlated). In addition, two miRNAs were associated with BMD of FN and TH (mir-330-5p was positively correlated, whilemir-3124-3p was negatively correlated). Signaling pathway enrichment for target genes of these five miRNAs were investigated and the results showed that Wnt signaling pathway was the most enrichment pathway relating to bone metabolism and osteogenic differentiation as shown in **Table 3**. Hence, we further analyzed the potential role of these five miRNA candidates in Wnt signaling pathway and online bioinformatics tools as TargetScan and DAVID was applied to help to predict target genes that may be involved in Wnt signaling pathway (**Figure 4**). Wnt family members, frizzled class receptors and dishevelled segment polarity proteins in Wnt signaling pathway were found for more than 3 times as target genes of those 5 miRNAs (**Table 4**)

**Table 2:** Differently expressed miRNAs in serum exosomes related to BMD.

miRNA-name	L1-L4		FN		TH	
	R	*p*	R	*p*	R	*p*
hsa-mir-324-3p	0.511	0.001	0.403	0.009	0.353	0.020
hsa-mir-766-3p	0.408	0.008	0.451	0.004	0.372	0.015
hsa-mir-1247-5p	-0.365	0.017	-0.341	0.024	-0.348	0.022
hsa-mir-330-5p	0.268	0.006	0.355	0.020	0.338	0.025
hsa-mir-3124-3p	-0.205	0.122	-0.339	0.025	-0.298	0.044

LS, lumbar spine; FN, femoral neck; TH, total hip.

**Table 3:** Signaling pathway enrichment for target genes of miRNAs related to BMD.

Term	*p*-Value	Fold Enrichment
Regulation of actin cytoskeleton	1.37E-04	2.790967
**Wnt signaling pathway**	0.004861	2.682394
Estrogen signaling pathway	0.040353	2.492729
Ras signaling pathway	9.60E-04	2.456883
**Regulating pluripotency of stem cells**	0.014823	2.423734
Rap1 signaling pathway	0.003138	2.350288
Hippo signaling pathway	0.023872	2.247171
VEGF signaling pathway	0.027434	2.174344
Long-term depression	0.040105	2.148561
Calcium signaling pathway	0.029788	2.067991
PI3K-Akt signaling pathway	0.003639	1.967089
Cytokine-cytokine receptor interaction	6.07E-04	1.867280
Vascular smooth muscle contraction	0.036487	1.853134
MAPK signaling pathway	0.003362	1.721263
Endocytosis	0.039149	1.611420

**Table 4:** Predicted target genes of miRNAs involved in Wnt signaling pathway.

mir-324-3p	mir-776-3p	mir-1247-5p	mir-330-5p	mir-3124-3p
WNT8B	WNT10A	WNT9B	WNT2B	WNT10B
FZD2	FZD10	DVL3	FZD4	LRP6
CSNK1E	SENP2	CSNK2A2	DVL3	CXXC4
DVL1	VANGL1	APC2	PRKACA	CSNK2A1
RAC3	WNT5B	MYCBP2	APC2	GSK3B
	PRKCA	RND1	BTRC	CSNK1A1L
	NFATC2		DVL1	FZD1
			PPP3CB	WNT5A
				PLCB1
				PRKCA

Wnt, Wnt family member; FZD, frizzled class receptor; CSNK1E, casein kinase 1 epsilon; DVL, dishevelled segment polarity protein; RAC3, rho family, small GTP binding protein Rac3; SENP2, SUMO1/sentrin/SMT3 specific peptidase 2; VANGL1, VANGL planar cell polarity protein 1; PRKCA, protein kinase C alpha; NFATC2, nuclear factor of activated T-cells, cytoplasmic, calcineurin-dependent 2; CSNK2A, casein kinase 2, alpha prime polypeptide; APC2, adenomatosis polyposis coli 2; MYCBP2, MYC binding protein 2, E3 ubiquitin protein ligase; RND1, Rho family GTPase 1; BTRC, beta-transducin repeat containing E3 ubiquitin protein ligase; PPP3CB, protein phosphatase 3 catalytic subunit beta; LRP6:LDL receptor related protein 6; CXXC4:CXXC finger protein 4; GSK3B, glycogen synthase kinase 3 beta; CSNK1A1L, casein kinase 1 alpha 1 like; PLCB1, phospholipase C beta 1.

### MiRNA Candidates Relating to BMD Could Regulate ALP Activity in hBMSCs

**Figure 5:**
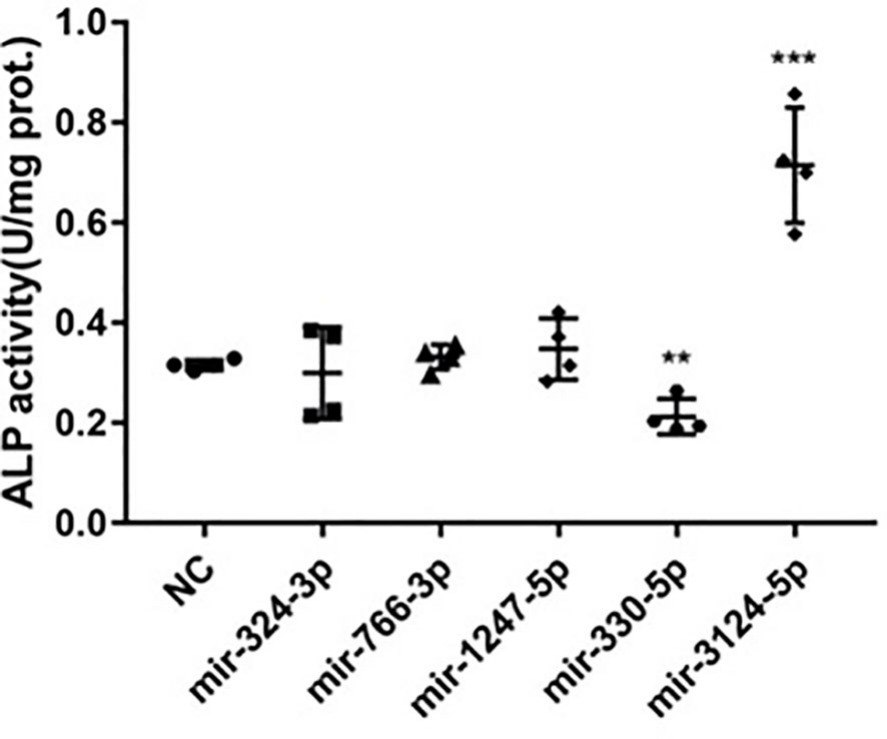
The function of miRNAs related to BMD in regulating ALP activity in hBMSCs. Five differently expressed miRNAs mimics (including mir-324-3p, mir-776-3p, mir-1247-5p,mir-330-5p and mir-3124-3p) associated with BMD were transfected into hBMSCs to upregulate correspondent miRNAs expression. ALP activity was examined on day 7 after transfection assay. Error bars represent SD of three independent experiments; **p < 0.01, ***p < 0.001.

Signaling pathway enrichment results showed that 5 miRNA candidates relating to BMD were also involved in regulating pluripotency of stem cells. To further confirm the function of these five miRNA candidates on bone turnover imbalance, ALP activity were detected in bone mesenchymal stem cells by transfecting with miRNA mimics or vehicles. ALP activity results showed that mir-330-5psuppressed ALP activity and inhibited the osteogenic differentiation of BMSCs, while mir-3124-3p showed the opposite result (**Figure 5**). In aggregate, these observations suggest that these differentially expressed miRNAs may be involved in the progression of PMOP and have potential to be novel diagnostic biomarkers of PMOP.

## Discussion

Osteoporosis and fracture have been strongly associated with women in post-menopausal age. Although clinical and basic research is constantly progressing, patients are still facing delayed diagnosis and fragility fractures, which indicate that exploration of circulating biomarkers is needed to provide a convenient and noninvasive diagnosis. MiRNAs are regarded as promising biomarkers to evaluate disease progression and miRNAs in the serum of OP patients has been investigated, and the patterns of circulating miRNAs are likely to be diagnostic predictors of OP (23). However, few data about miRNAs, let alone circulating exosomal miRNAs, are available in PMOP with fragility fractures. Hence, we presented, for the first time, the serum exosomal miRNAs expression profiles in postmenopausal women, and compared the differences between women suffered from severe osteoporosis and those with normal BMD and discovered exosome miRNAs with promising diagnostic values. In the DEGseq, among miRNAs with fold change >2 and p-value<0.5, 169miRNAs were found to be significantly upregulated, while 70miRNAs were downregulated in SOP group, compared to CON group. GO functional analysis was applied to describe the items of BP, CC and MF terms that provided significant clues to studying molecular functions in the progression of osteoprosis.

PMOP with fragility fractures is a complex biological process that involves complicated signaling pathways. In this study, we focused on miRNAs associated with BMD and related molecular mechanisms to gain insight into the link between PMOP with fragility fractures and miRNAs. We found five exosomal miRNAs (mir-324-3p, mir-766-3p, mir-1247-5p, mir-330-5p and mir-3124-5p) were related to BMD. Moreover, predicted target genes of these five miRNAs were highly associated with Wnt signaling pathway. Wnt signaling pathway is well known for its role in regulating self-renewal and differentiation in stem cells and bone metabolism (24–27). Wnt family members, frizzled class receptors and dishevelled segment polarity proteins in Wnt signaling pathway were notable as target genes of those 5 miRNAs that may provide rewarding points for further research. Among them, mir-324-3p, mir-766-3p and mir-1247-5p were found to be associated with BMD of the lumbar spine, femoral neck and hip sites, while mir-330-5p and mir-3124-5p were found to be associated with BMD of the hip. Previous studies (28) proved that mir-324-3p was expressed at low levels in low-traumatic fractures, indicating that in elderly individuals, low expression of mir-324-3p may result in fractures by reducing bone density. Mir-766-3p could reduce the protein expression of Wnt3a (29) and NF-κB (30), which play important roles in OP. In breast tumors, mir-1247-5p promotes tumor growth *via* the Dishevelled1(DVL1)/Wnt/β-catenin signaling pathway (31), which promotes the differentiation of skeletal cells and accelerates bone regeneration (32). However, not for all the five miRNAs found associated with BMD, the function in bone metabolism has been validated with *in vitro* study. The only two miRNAs found involved in ALP activity are mir 330-5p and mir- 3124 with the highest fold change (mir-330-5p [log_2_FC=-5.84], mir-3124-5p [log_2_FC=5.72]). Mir-330-5p suppress ALP activity and inhibit the osteogenic differentiation of BMSCs, and mir-3124-5p significantly promoted osteogenic differentiation of BMSCs. Mir-330-5p was reported to be upregulated in senescent MSCs compared with young MSCs (33). MSCs are known to have self-renewal and multi-differentiation abilities, and a reduction in osteogenic differentiation of MSCs leads to loss of bone mass and contributes to increased risk of fracture. Knockdown of mir-330-5p facilitates osteogenesis through the biglycan-induced bone morphogenetic protein (BMP)/Smad pathway and further to influence the progression of OP (34). On the other hand, mir-330-5p was found to silence SPRY2 expression and further influence the progression of tumors *via* Mitogen-activated protein kinases/extracellular signal-regulated kinase (MAPK/ERK) signaling (35), which is a regulator of osteoclastogenesis and plays an important role in bone loss (36). In our study, mir-330-5p in exosomes was positively correlated to the BMD of FN and TH and *in vitro* suppress ALP activity and inhibit the osteogenesis. These results indicate that for those people with relatively high bone mass, the expression of mir-330-5p may be elevated to suppress the osteogenic differentiation of MSCs to maintain the balance of bone metabolism. There were few studies on mir-3124-5p before, however, in this study we found mir-3124-5p was negatively related to BMD *in vivo*, and significantly promoted osteogenesis *in vitro*. We consider that in patients with low bone mass, the expression of mir-3124-5p may be upregulated compensatively to promote the osteogenic differentiation of MSCs to prevent bone loss. These results suggested that the exosomal miRNAs candidates associated with BMD might play complex regulatory roles in the progression of PMOP by networking with cell signaling pathways, and mir-330-5p and mir-3124-5p in circulating exosomes could not only be biomarkers but also functional molecules in the progression of PMOP. However, more studies are needed to clarify the molecular mechanisms of miRNAs in circulating exosomes in PMOP with fragility fractures.

In conclusion, this study provided the first information on differential serum exosomes miRNA expression profiling between severe osteoporosis and normal BMD in postmenopausal women using second-generation sequencing. mir-324-3p, mir-766-3p, mir-1247-5p,mir-330-5p and mir-3124-5p were found to be associated with BMD, but only miR-330 and miR-3124 had been confirmed its role in bone metabolism *in vitro*, which may serve as circulating biomarkers as well as therapeutic targets and treatment options for PMOP. However, in order to apply these miRNA profiles in clinical practice, further studies on prospectively collected datasets are needed to validate these findings, and more reliable and reproducible analysis model are required in following studies.

## Data Availability Statement

The original contributions presented in the study are publicly available. They can be found on Figshare via the DOI 10.6084/m9.figshare.17086307.

## Ethics Statement

The studies involving human participants were reviewed and approved by Medical Ethics Committee of Huadong Hospital (2019K055). The patients/participants provided their written informed consent to participate in this study.

## Author Contributions

QC designed the study. HS and QC drafted the manuscript. QC, XJ, and HS contributed to its refinement. QC and HS recruited the patients and collected the data. HS, XJ, and CX performed the statistical analysis. QC, HS, and XJ interpreted the analytical data. All authors contributed to the article and approved the submitted version.

## Funding

The research was funded by grants from the National Key Research and Development Program of China (2018YFC2000203); National Natural Science Foundation of China (NSFC; No. 81471089); Shanghai Municipal Health Bureau (GWV-9.4).

## Conflict of Interest

The authors declare that the research was conducted in the absence of any commercial or financial relationships that could be construed as a potential conflict of interest.

## Publisher’s Note

All claims expressed in this article are solely those of the authors and do not necessarily represent those of their affiliated organizations, or those of the publisher, the editors and the reviewers. Any product that may be evaluated in this article, or claim that may be made by its manufacturer, is not guaranteed or endorsed by the publisher.

## Supplementary Material

Supplementary Figure 1 Size distribution and concentration difference of exosomes in two groups were analyzed by the Nanoparticle tracking analysis.
