# Supplementary material for: MicroRNAs in Serum Exosomes as Circulating Biomarkers for Postmenopausal Osteoporosis
Source: Front Endocrinol (Lausanne). 2022 Mar 10;13:819056. doi: 10.3389/fendo.2022.819056 (PMC8960856; doi:10.3389/fendo.2022.819056)
Supplement: Supplementary Figure 1 — Size distribution and concentration difference of exosomes in two groups were analyzed by the Nanoparticle tracking analysis. [file DataSheet_1.docx]

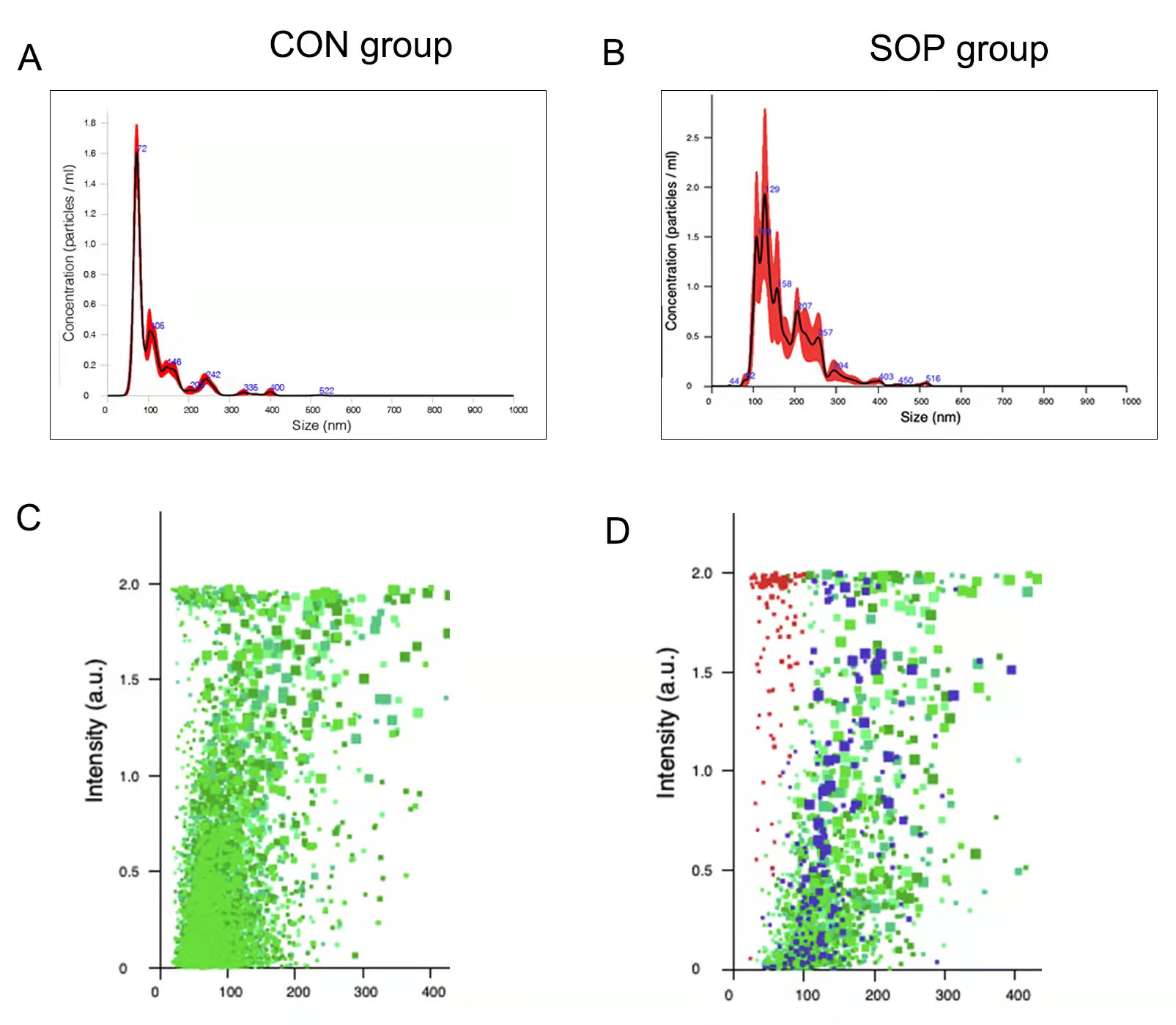
**Supplementary Figure1.Size distribution and concentration difference of exosomes in two groups were analysed by the nanoparticle tracking analysis.**
