# Supplementary material for: MicroRNAs in Serum Exosomes as Circulating Biomarkers for Postmenopausal Osteoporosis
Source: Front Endocrinol (Lausanne). 2022 Mar 10;13:819056. doi: 10.3389/fendo.2022.819056 (PMC8960856; doi:10.3389/fendo.2022.819056)
Supplement: Supplementary file 2 [file Table_1.pdf]

Supplementary Table 1. Differentially expressed miRNAs of the 2 groups by small RNA sequencing.

miRNA id	log2Ratio(B/A)	Up/down	P value
hsa-miR-1181	-3.78916	DOWN	0.000328
hsa-miR-1224-5p	-5.15173	DOWN	1.22E-05
hsa-miR-1229-5p	-4.88869	DOWN	7.33E-05
hsa-miR-1231	-4.37412	DOWN	4.44E-06
hsa-miR-1246	-1.10298	DOWN	0
hsa-miR-1269b	-4.15173	DOWN	0.003039
hsa-miR-1279	-3.78916	DOWN	0.000328
hsa-miR-1284	-3.83978	DOWN	2.06E-07
hsa-miR-1290	-2.44006	DOWN	0
hsa-miR-1301-3p	-1.9818	DOWN	3.77E-05
hsa-miR-145-5p	-2.60983	DOWN	2.24E-11
hsa-miR-152-3p	-1.8183	DOWN	0.001189
hsa-miR-1537-3p	-4.62566	DOWN	3.99E-07
hsa-miR-1908-3p	-4.50536	DOWN	1.33E-06
hsa-miR-1911-3p	-4.68224	DOWN	0.000248
hsa-miR-199a-5p	-1.01967	DOWN	6.62E-09
hsa-miR-200a-3p	-1.68875	DOWN	8.72E-11
hsa-miR-200b-3p	-3.30373	DOWN	6.12E-07
hsa-miR-200c-3p	-2.3323	DOWN	5.00E-21
hsa-miR-29b-3p	-1.44123	DOWN	0.001626
hsa-miR-30d-3p	-3.22973	DOWN	8.48E-05
hsa-miR-31-5p	-1.37331	DOWN	2.83E-15
hsa-miR-3181	-4.15173	DOWN	2.77E-05
hsa-miR-3188	-6.02619	DOWN	2.63E-09
hsa-miR-324-3p	-1.54252	DOWN	9.37E-06
hsa-miR-330-5p	-5.84123	DOWN	0.000248
hsa-miR-342-5p	-2.15173	DOWN	9.06E-09
hsa-miR-345-5p	-1.49249	DOWN	4.93E-20
hsa-miR-3605-5p	-5.37412	DOWN	2.12E-06
hsa-miR-3661	-5.15173	DOWN	1.22E-05
hsa-miR-3679-3p	-4.68224	DOWN	0.000248
hsa-miR-3679-5p	-5.44123	DOWN	6.48E-12

hsa-miR-369-3p	-1.78916	DOWN	0.003303
hsa-miR-378d	-7.35684	DOWN	5.59E-20
hsa-miR-4287	-3.44123	DOWN	0.002129
hsa-miR-4433b-3p	-2.28136	DOWN	2.02E-17
hsa-miR-4443	-3.02619	DOWN	4.97E-07
hsa-miR-4456	-4.44123	DOWN	0.000857
hsa-miR-4492	-1.9818	DOWN	0.000279
hsa-miR-451a	-1.59355	DOWN	0
hsa-miR-4640-3p	-4.62566	DOWN	3.99E-07
hsa-miR-4732-3p	-1.9818	DOWN	7.31E-07
hsa-miR-4745-5p	-5.73669	DOWN	7.06E-08
hsa-miR-4772-3p	-5.50536	DOWN	6.73E-07
hsa-miR-4787-5p	-1.11108	DOWN	0.000179
hsa-miR-485-3p	-2.12316	DOWN	5.02E-10
hsa-miR-493-3p	-2.85627	DOWN	6.60E-05
hsa-miR-548ae-5p	-5.30373	DOWN	3.79E-06
hsa-miR-550a-3-5p	-4.15173	DOWN	0.003039
hsa-miR-6089	-2.30373	DOWN	7.92E-06
hsa-miR-616-3p	-5.37412	DOWN	2.12E-06
hsa-miR-6165	-5.73669	DOWN	7.06E-08
hsa-miR-651-5p	-4.15173	DOWN	0.003039
hsa-miR-654-5p	-2.44123	DOWN	7.18E-06
hsa-miR-6716-5p	-1.39684	DOWN	6.09E-05
hsa-miR-6727-5p	-4.44123	DOWN	0.000857
hsa-miR-6731-3p	-5.50536	DOWN	6.73E-07
hsa-miR-6741-5p	-1.34745	DOWN	1.98E-05
hsa-miR-6743-5p	-1.0945	DOWN	1.89E-16
hsa-miR-6763-5p	-2.45573	DOWN	4.51E-15
hsa-miR-6769a-3p	-4.15173	DOWN	0.003039
hsa-miR-6775-5p	-2.072	DOWN	4.57E-16
hsa-miR-6836-3p	-1.44264	DOWN	1.59E-18
hsa-miR-7107-5p	-2.9818	DOWN	2.07E-05
hsa-miR-7156-3p	-5.50536	DOWN	6.73E-07
hsa-miR-766-3p	-1.37815	DOWN	1.29E-12

hsa-miR-7855-5p	-1.75941	DOWN	0.001905
hsa-miR-7975	-6.30373	DOWN	6.41E-11
hsa-miR-877-5p	-2.62006	DOWN	1.40E-29
hsa-miR-942-3p	-3.9818	DOWN	9.49E-05
hsa-let-7c-5p	1.640044	UP	0
hsa-let-7e-3p	1.075533	UP	0.000117
hsa-miR-101-3p	3.266127	UP	7.05E-05
hsa-miR-103a-2-5p	4.188125	UP	0.002792
hsa-miR-106b-3p	2.603162	UP	0.000543
hsa-miR-1178-5p	3.718639	UP	0.000538
hsa-miR-1227-5p	2.410517	UP	0.000348
hsa-miR-1228-3p	4.188125	UP	0.002792
hsa-miR-1229-3p	3.105663	UP	0.000237
hsa-miR-1236-5p	4.92509	UP	6.48E-05
hsa-miR-1238-5p	5.92509	UP	1.10E-08
hsa-miR-124-3p	1.85219	UP	2.20E-09
hsa-miR-1247-5p	2.340128	UP	0.002971
hsa-miR-1249-3p	1.650468	UP	1.13E-11
hsa-miR-125b-1-3p	7.493933	UP	2.38E-21
hsa-miR-125b-5p	1.362416	UP	1.51E-53
hsa-miR-1292-3p	2.437152	UP	2.99E-18
hsa-miR-129-2-3p	5.477631	UP	1.01E-06
hsa-miR-1292-5p	4.718639	UP	0.000222
hsa-miR-129-5p	9.606914	UP	7.21E-69
hsa-miR-1297	4.477631	UP	0.000777
hsa-miR-130a-3p	1.480543	UP	3.57E-10
hsa-miR-135a-5p	2.340128	UP	1.39E-10
hsa-miR-138-5p	2.255239	UP	1.64E-05
hsa-miR-139-3p	1.269889	UP	1.00E-51
hsa-miR-140-3p	4.340128	UP	0.001468
hsa-miR-148a-3p	1.400669	UP	6.66E-11
hsa-miR-149-3p	2.477631	UP	0.0002
hsa-miR-149-5p	7.479679	UP	0.000067
hsa-miR-153-3p	4.972396	UP	8.89E-09

hsa-miR-17-5p	1.121293	UP	1.01E-06
hsa-miR-181b-5p	1.540084	UP	1.02E-11
hsa-miR-181c-5p	1.256604	UP	1.02E-09
hsa-miR-181d-5p	1.908971	UP	2.01E-10
hsa-miR-1827	4.477631	UP	0.000777
hsa-miR-186-5p	2.972396	UP	1.16E-06
hsa-miR-187-3p	7.469411	UP	1.88E-40
hsa-miR-18a-5p	2.956799	UP	3.48E-09
hsa-miR-1908-5p	1.288289	UP	8.65E-11
hsa-miR-193a-5p	2.214597	UP	7.22E-15
hsa-miR-195-5p	1.487685	UP	1.62E-06
hsa-miR-199a-3p	1.257185	UP	1.12E-10
hsa-miR-204-3p	4.510053	UP	9.86E-12
hsa-miR-206	1.603162	UP	0.000109
hsa-miR-212-3p	1.935738	UP	2.00E-08
hsa-miR-215-5p	1.405223	UP	0.001588
hsa-miR-218-5p	1.027786	UP	2.03E-07
hsa-miR-219a-2-3p	8.137141	UP	1.10E-30
hsa-miR-219a-5p	4.477631	UP	2.00E-06
hsa-miR-223-5p	1.088589	UP	0.000435
hsa-miR-22-3p	1.004268	UP	0
hsa-miR-22-5p	2.734407	UP	1.37E-08
hsa-miR-23a-5p	3.718639	UP	9.86E-07
hsa-miR-24-3p	1.412973	UP	0
hsa-miR-25-5p	4.541762	UP	5.42E-12
hsa-miR-27b-5p	1.92509	UP	0.000204
hsa-miR-296-5p	1.152849	UP	3.09E-06
hsa-miR-29a-3p	2.324303	UP	0.000017
hsa-miR-29b-1-5p	4.188125	UP	0.002792
hsa-miR-29c-3p	1.244971	UP	2.29E-07
hsa-miR-302c-5p	2.603162	UP	1.38E-15
hsa-miR-3074-5p	1.32192	UP	6.28E-89
hsa-miR-30e-5p	1.802471	UP	0.000305
hsa-miR-3124-5p	5.718639	UP	1.03E-07

hsa-miR-3141	1.496247	UP	0.000393
hsa-miR-3163	5.0182	UP	3.53E-05
hsa-miR-3180	4.603162	UP	0.000414
hsa-miR-3187-3p	2.681165	UP	3.28E-07
hsa-miR-3189-3p	2.266127	UP	3.52E-06
hsa-miR-320a	1.053553	UP	4.55E-99
hsa-miR-320b	1.681165	UP	0.00014
hsa-miR-320d	1.713909	UP	7.24E-54
hsa-miR-320e	6.900843	UP	3.20E-15
hsa-miR-323a-3p	2.043735	UP	2.93E-20
hsa-miR-323a-5p	3.603162	UP	0.001013
hsa-miR-326	2.141582	UP	1.39E-08
hsa-miR-335-5p	1.551632	UP	1.53E-05
hsa-miR-338-5p	1.935738	UP	7.24E-05
hsa-miR-34a-5p	2.215161	UP	1.53E-09
hsa-miR-34b-3p	2.88327	UP	8.11E-10
hsa-miR-34c-3p	4.92509	UP	6.48E-05
hsa-miR-3529-3p	1.399803	UP	2.21E-43
hsa-miR-3615	1.433237	UP	8.80E-07
hsa-miR-361-5p	2.0182	UP	7.22E-12
hsa-miR-3617-5p	5.773087	UP	5.83E-08
hsa-miR-363-3p	1.0182	UP	1.56E-05
hsa-miR-3656	1.507324	UP	0
hsa-miR-365a-3p	2.718639	UP	0.002622
hsa-miR-365b-3p	4.541762	UP	5.42E-12
hsa-miR-369-5p	1.008374	UP	2.71E-18
hsa-miR-371a-5p	5.541762	UP	5.68E-07
hsa-miR-374b-3p	2.105663	UP	0.00304
hsa-miR-374c-3p	2.427591	UP	4.99E-13
hsa-miR-375	1.097859	UP	7.77E-44
hsa-miR-378e	1.94605	UP	1.46E-17
hsa-miR-378f	6.227653	UP	3.47E-19
hsa-miR-378i	3.628925	UP	5.67E-91
hsa-miR-381-3p	3.92509	UP	0.000153

hsa-miR-421	4.92509	UP	6.48E-05
hsa-miR-423-5p	1.125115	UP	4.97E-13
hsa-miR-4267	4.188125	UP	0.002792
hsa-miR-4297	3.603162	UP	0.001013
hsa-miR-4313	3.825555	UP	0.000287
hsa-miR-431-5p	1.0182	UP	3.93E-05
hsa-miR-4417	2.92509	UP	0.000793
hsa-miR-4442	6.477631	UP	6.16E-12
hsa-miR-4451	6.825555	UP	1.41E-14
hsa-miR-4455	4.718639	UP	0.000222
hsa-miR-449a	9.026628	UP	0
hsa-miR-4508	3.477631	UP	7.59E-08
hsa-miR-4535	2.532773	UP	3.11E-24
hsa-miR-454-3p	1.313656	UP	3.66E-06
hsa-miR-455-5p	4.0182	UP	8.19E-05
hsa-miR-4659a-3p	6.541762	UP	2.19E-12
hsa-miR-4687-5p	5.773087	UP	5.83E-08
hsa-miR-4706	4.477631	UP	0.000777
hsa-miR-4787-3p	4.188125	UP	0.002792
hsa-miR-4802-5p	4.603162	UP	0.000414
hsa-miR-487a-5p	4.0182	UP	8.19E-05
hsa-miR-499a-5p	1.583797	UP	6.03E-13
hsa-miR-512-5p	2.0182	UP	7.60E-05
hsa-miR-513c-5p	5.0182	UP	3.53E-05
hsa-miR-516a-5p	4.477631	UP	0.000777
hsa-miR-5193	2.340128	UP	0.000603
hsa-miR-519e-5p	3.92509	UP	0.000153
hsa-miR-532-3p	6.188125	UP	4.18E-10
hsa-miR-550b-3p	5.340128	UP	3.25E-06
hsa-miR-5585-3p	5.188125	UP	1.27E-18
hsa-miR-572	1.0182	UP	0.002477
hsa-miR-589-3p	5.876181	UP	1.91E-08
hsa-miR-6084	2.477631	UP	0.0002
hsa-miR-6088	1.396711	UP	0.000761

hsa-miR-629-3p	1.634871	UP	4.01E-05
hsa-miR-634	6.572789	UP	1.31E-12
hsa-miR-6509-5p	4.603162	UP	0.000414
hsa-miR-6513-3p	4.972396	UP	8.89E-09
hsa-miR-671-5p	2.296735	UP	3.91E-18
hsa-miR-6722-3p	5.541762	UP	5.68E-07
hsa-miR-6729-3p	2.730918	UP	1.32E-10
hsa-miR-6749-5p	1.699378	UP	1.94E-09
hsa-miR-6752-5p	5.105663	UP	1.93E-05
hsa-miR-675-3p	2.266127	UP	0.00104
hsa-miR-6766-3p	4.825555	UP	0.00012
hsa-miR-6789-3p	2.340128	UP	0.000603
hsa-miR-6798-3p	5.972396	UP	6.31E-09
hsa-miR-6805-3p	3.603162	UP	0.001013
hsa-miR-6824-3p	5.340128	UP	3.25E-06
hsa-miR-6829-3p	5.105663	UP	1.93E-05
hsa-miR-6842-3p	3.105663	UP	0.000237
hsa-miR-6865-5p	1.298308	UP	2.61E-06
hsa-miR-6889-5p	4.188125	UP	0.002792
hsa-miR-6891-5p	5.266127	UP	5.86E-06
hsa-miR-7159-5p	5.105663	UP	1.93E-05
hsa-miR-760	3.755165	UP	1.12E-09
hsa-miR-766-5p	5.188125	UP	1.06E-05
hsa-miR-767-3p	6.266127	UP	1.43E-10
hsa-miR-769-3p	3.603162	UP	0.001013
hsa-miR-7845-5p	4.188125	UP	0.002792
hsa-miR-8072	2.647556	UP	2.69E-16
hsa-miR-877-3p	4.340128	UP	0.001468
hsa-miR-885-5p	1.403693	UP	3.10E-20
hsa-miR-92a-1-5p	4.92509	UP	6.48E-05
hsa-miR-92b-3p	2.808916	UP	0
hsa-miR-92b-5p	1.688051	UP	0.000247
hsa-miR-935	2.410517	UP	0.000348
hsa-miR-937-5p	6.105663	UP	1.23E-09

hsa-miR-9-3p	7.913017	UP	1.38E-05
hsa-miR-9-5p	5.680246	UP	0
hsa-miR-99a-5p	1.173668	UP	0
